# Genomic traits associated with copiotrophy decouple from maximum growth rate predictions along temperature gradients

**DOI:** 10.1093/ismejo/wrag147

**Published:** 2026-06-09

**Authors:** J L Weissman, Alexandra Walling, Hugh Ducklow, Emily J Zakem

**Affiliations:** Institute for Advanced Computational Science, Stony Brook University, Stony Brook, NY 11794, United States; Department of Ecology & Evolution, Stony Brook University, Stony Brook, NY 11794, United States; Institute for Advanced Computational Science, Stony Brook University, Stony Brook, NY 11794, United States; Department of Ecology & Evolution, Stony Brook University, Stony Brook, NY 11794, United States; Lamont Doherty Earth Observatory, Columbia University, Palisades, NY 10964, United States; Biosphere Sciences and Engineering, Carnegie Institution for Science, Pasadena, CA 91125, United States

**Keywords:** Copiotrophy, Microbial Growth, Optimal Growth Temperature, Genomics, Maximum Growth Rate

## Abstract

Maximum growth rate is often used as a primary axis of functional variation in studies of microorganisms, in part because emerging tools make it straightforward to estimate from genomic and metagenomic data. However, temperature, via its influence on reaction kinetics, may act as a confounder in studies that measure genomic signatures of growth optimization across environments. Observations suggest that growth optimization need not always indicate rapid growth. For example, strong temperature gradients are the norm across much of the world’s oceans, where deep-ocean microbes show elevated signals of genomic growth optimization relative to the faster-growing communities at the surface. Looking across environments, we find a negative relationship between genomic growth optimization and optimal growth temperature, leading to the potential decoupling of genomic traits associated with copiotrophy from maximum growth rate, particularly when measured along a temperature gradient. Our results suggest that, as a result of temperature’s confounding effects, genomic signatures of growth optimization often better predict the ecological roles and functional genomic content of microorganisms than do growth rates themselves. Finally, we suggest reframing copiotrophy as growth beyond a thermodynamic baseline maximum growth rate, rather than in relation to a static rate cutoff.

## Introduction

A persistent problem for linking microbial process to global-scale biogeochemical cycling in both terrestrial and aquatic environments is that microbial communities are incredibly functionally and taxonomically diverse, often comprising many hundreds or thousands of species [1–3]. Genomic trait inference, wherein microbial phenotypes are estimated directly from genomic and metagenomic data, provides a path forward to systematically organize microbial functional diversity [4, 5]. For example, estimating maximum growth rate from genomes groups organisms from across the microbial tree of life into fast- and slow-growing clusters with distinct evolutionary patterns and genomic functional content [6, 7]. Genomically inferred growth strategies have also recently been shown to align closely with the global-scale biogeography and functional roles of marine picoheterotrophs [8].

Maximum growth rate is often included as a genomically tractable axis of functional variation in comparative studies [6, 9], because variation in maximum growth rate is conceptually related to the “copiotrophy-oligotrophy” axis used by many to organize microbial functional roles within ecosystems. Under this framework, fast-growing “copiotrophs” are optimized for high-resource environments and slow-growing “oligotrophs” are optimized for low-resource environments [7, 10]. Specialization for environments in which resources are abundant versus scarce need not necessarily align directly with maximum growth rate [6], because other parameters also matter for such fitness [11, 12], but fundamental rate-yield tradeoffs in ATP production pathways suggest that these two features of microbial lifestyle (resource abundance specialization and maximum growth rate) should often be correlated [13, 14], and indeed metabolic traits associated with canonical “copiotrophs” also tend to be associated with fast growth [6, 7]. Other trait-based frameworks for characterizing microbial diversity include additional axes like stress-response, but still emphasize a core tradeoff between growth rate (or “resource acquisition”) and efficiency of resource utilization that maps onto the copiotroph-oligotroph dichotomy [15, 16].

Genomic approaches allow for direct estimation of microbial maximum growth rate and thus potentially facilitate placement of organisms along the copiotrophy-oligotrophy axis [6, 9]. Several genomic features thought to be associated with optimization for rapid translation have also been shown to be associated with rapid growth, including elevated codon usage bias of highly expressed genes, high rRNA and tRNA gene copy numbers, increased translation initiation start sites, and the presence of elongation factor P and a reduction in polyproline sequences (Supplementary Fig. S1A–C; [9, 14, 17, 18]). Each of these features is associated with a specific mechanistic hypothesis (e.g. that codon optimization allows for rapid translation which allows for faster biomass production, that polyproline sequences stall translation and thus slow down this process without the assistance of elongation factor P). More broadly, translation optimization can be thought of as an indicator of high ribosomal investment over evolutionary time, which should correlate with maximum growth rate according to fundamental laws of bacterial growth [19, 20]. These features, most commonly codon usage bias and rRNA copy number, are frequently used in statistical models that predict the maximum growth rate of an organism from its genome sequence [6, 9, 14].

This simple picture in which growth rate and preferred resource environment are linked is complicated by the fact that factors unrelated to resource abundance can exert substantial control on absolute maximum growth rates, including temperature [9, 21] and substrate “quality” (here, considering lability) [8], in ways that are orthogonal to the classical copiotrophy-oligotroph spectrum of substrate availability [8]. For example, temperature strongly constrains growth through basic thermodynamics; reactions proceed faster at higher temperatures, barring enzyme denaturation. Thus, temperature constrains microbial growth such that organisms with clear patterns of genomic optimization for rapid growth in relatively colder environments will in reality grow much more slowly than organisms showing no such patterns of optimization that live in warmer environments. Looking across sequenced microbial genomes, accurate estimates of an organism’s maximum growth rate on the basis of genomic data require an estimate of optimal growth temperature [6, 9]. Similarly, if the metabolism of an organism produces relatively little ATP (e.g. methanogenesis), even a great deal of translation optimization may not increase growth rates to the levels seen in organisms with more energetically favorable metabolic pathways. In other words, copiotrophy need not always mean fast growth. One example of this decoupling comes from considering a gradient in the “quality” of organic substrates fueling heterotrophic microbial growth, where in marine systems it has been shown that organisms living deep in the water column, believed to be consuming abundant but recalcitrant (low quality) carbon sources, have enhanced genomic signals of translation optimization even though their maximum growth rates are necessarily very low [8]. This slow growth reflects the less-favorable energetics of the metabolisms of these organisms as well as the fact that these organisms live in cold environments [8, 22]. Temperature in particular is a potential confounder across diverse study systems, especially given the increase in research interest studying organismal responses to a warming climate.

Here, we address how temperature gradients can confound inferences made about the evolution of a microbial population’s life history strategy (i.e. copiotrophy) from maximum growth rates. We demonstrate that over gradients in temperature, genomic correlates of maximum growth rate, such as the degree of codon usage bias across an organism’s highly expressed genes [9, 22], may, in fact, tell us more about the ecology of an organism than the maximum growth rate itself. In particular, we demonstrate that potentially misleading interpretations of maximum growth rate may be gleaned when there exists a negative correlation between organismal optimal growth temperatures and genomic signatures of translation rate optimization. We additionally show that such a relationship between these two traits is the norm rather than an exception. In such situations (e.g. along temperature gradients), we suggest that ecological patterns are better recovered by genomic indices of growth optimization, rather than direct growth rate measurements, whether maximal or instantaneous, in line with recent work showing that these two pieces of information about an organism do not always align in the environment [23].

## Materials and methods

For all bioinformatics analyses, default parameters were used unless otherwise specified. All data and scripts used to generate analyses and figures available at https://github.com/jlw-ecoevo/copio_temp/. For model details, analysis, and a summary of data sources used in each figure panel see supplement (Supplementary Text S1, Supplementary Table S1).

### Genomic trait database annotation

We obtained the 113 104 species-level representative genomes from the GTDB v220 release, which includes a combination of genomes sequenced from isolates, high-quality metagenome-assembled genomes (MAGs), and high-quality single-cell amplified genomes (SAGs) [24–27]. All of these genomes were annotated using prokka v1.14.6 (using “--kingdom Archaea” for archaeal genomes) [28]. Carbohydrate-active enzymes were annotated using dbCan v2.0.11 with annotations retained if confirmed by at least two methods [29]. Optimal growth temperatures were predicted using GenomeSPOT v1.0.1 [30]. GenomeSPOT has high accuracy for *T*_opt_ prediction (*R*^2^ > 0.7), and is able to confidently differentiate thermophiles and mesophiles, similar to other genomic *T*_opt_ predictors which readily identify thermophiles but struggle to make predictions for psychrophilic organisms [31]. Maximum growth rates and codon statistics were estimated using gRodon v2.4.0 for the 112 441 genomes with sufficient numbers of annotated ribosomal proteins for confident prediction ([6, 22]; using metagenome mode to control for any potential contamination in the MAGs and SAGs and using temperature predictions from GenomeSPOT). Species-level 16S rRNA gene counts were obtained from rrnDB-5.9 and mapped to 4357 GTDB v220 representative genomes by species name [32]. For 12 553 species representative genomes we were additionally able to match experimentally measured optimal growth temperatures in the Gosha database which are drawn from the literature [33]. A subset of one randomly selected representative genome per family (5463 genomes) was also annotated using eggnogmapper v2.1.12 [34] and resulting gene families were grouped into pathways using KEGG-Decoder and KEGGaNOG v1.1.17 [35].

## Other trait data

Genome assemblies for the 20 northern and southern phylogroup genomes described by Choudoir *et al.* [36] were annotated with predicted maximum growth rates and optimal growth temperatures using gRodon v2.4.0 [6] and GenomeSPOT v1.0.1 [30].

We also re-analyzed the original 17 347 genomes matched to 444 species with experimentally measured maximum growth rates and optimal growth temperatures from the literature taken from the Madin *et al.* [37] trait database that was used to train the original gRodon software.

BIOGEOTRACES and Malaspina metagenomes [38, 39] were annotated with maximum growth rates as described in Weissman *et al.* [22]. Temperatures were taken from BIOGEOTRACES and Malaspina metadata, respectively [38, 39].

Leucine uptake data from the AESOPS cruises were obtained from BCO-DMO [40], and data from the Malaspina Expedition were also retrieved [41]. For the AESOPS data, bulk leucine incorporation rates were divided by cell counts to obtain per-cell rate data. For the Malaspina data, measurements were made directly on individual cells, obviating the need for normalization.

Surface temperature data were obtained from NASA MODIS [42, 43] and body temperature data from Moreira *et al.* [44].

### Statistical analysis

We performed phylogenetically controlled linear regression analyses using a Brownian motion model in the phylolm v2.6.5 R package [45] and the phylogeny of representative species genomes provided with the GTDB v220 release. The robustness of phylogenetic regressions to specific species and phyla, and to sample size, were assessed using the sensiphy v0.8.5 R package [46] which repeatedly drops data from the dataset to assess sensitivity.

The growth model in Equation 2 was fit to the original gRodon training data of maximum growth rates and optimal growth temperatures from the Madin *et al.* [37] trait database using multiple regression (lm in R v4.3.3 base stats). The diminishing returns model (described in Supplementary Text S1) was fit to the data using the nls function in R v4.3.3 base stats.

## Results and discussion

### Modeling growth optimization as divergence from a thermodynamic baseline

**Figure 1:**
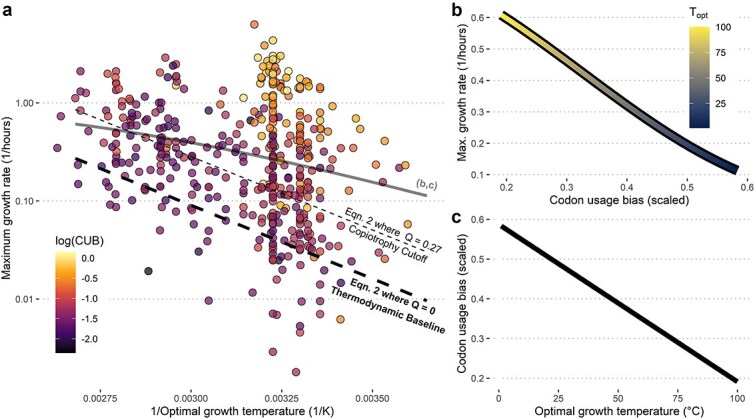
The growth rate versus translation optimization relationship can reverse along temperature gradients where translation optimization and optimal growth temperature are anti-correlated. (a) Maximum growth rate and *T*_opt_ data for species taken from the literature with scaled codon usage bias of the ribosomal proteins for corresponding genomes overlaid. Dashed lines show the fit of the modified Arrhenius model from Equation 2 for the theoretical maximum growth rate versus 1/*T*_opt_ relationship for organisms without any translation optimization (scaled CUB of zero, i.e. *Q* = 0) as well as for organisms at the hypothetical oligotrophy/copiotrophy cutoff (scaled CUB of 0.27; based on clustering of species in Weissman *et al.* [6]). (b and c) Following Equation 2, if we sample a series of organisms along a range of *T*_opt_ for which codon usage bias (*Q*) and *T*_opt_ are inversely related, it is possible that codon usage bias and maximum growth rate will also be negatively related across these organisms. The line shown in (b and c) corresponds to the solid line in panel (a).

Maximum growth rates are expected to increase with optimal growth temperature (*T*_opt_) [9, 47–49], and this pattern is also represented in commonly used statistical models for maximum growth rate prediction. Together, genomic maximum growth rate prediction tools [6, 9] generally use the following, or very similar (e.g. with a Box-Cox transformation in place of a log transformation), model for maximum growth rate (${\mu}_{\mathrm{max}}$):


(1) \begin{eqnarray*} {\mu}_{\mathrm{max}}={e}^{\beta_0+{\beta}_1{T}_{opt}+{\beta}_2Q} \end{eqnarray*}

where *Q* is some measure of translation rate optimization (typically codon usage bias or ribosomal gene copy number) and the coefficients ${\beta}_i$ are fit using data drawn from the literature or experiments. Alternatively, we introduce a minor modification that turns this model into the Arrhenius curve:


(2) \begin{eqnarray*} {\mu}_{\mathrm{max}}={e}^{\beta_0+{\beta}_1\left({\frac{1}{T}}_{opt}\right)+{\beta}_2Q}={e}^{\beta_0+{\beta}_2Q}{e}^{{\frac{\beta_1}{T}}_{opt}}=A{e}^{-\frac{E_b}{RT}} \end{eqnarray*}

where $A={e}^{\beta_0+{\beta}_2Q}$ is the pre-exponential factor, representing the speed of a reaction mechanism (the frequency of successful collisions of reactants), ${\beta}_1=-{E}_b/R$, ${E}_b$ is the activation energy of the reaction, representing the minimum energy input to initiate the reaction, and *R* is the universal gas constant. Conceptually, *A* gives us the fundamental speed of the mechanism of growth, whereas ${e}^{{\frac{\beta_1}{T}}_{\mathrm{opt}}}$ gives the temperature dependence of growth in units of Kelvin. This alternative model fits experimental growth and temperature data slightly better than Equation 1 (gRodon [6] training data, see Methods; linear regression with a log transform, Equation 1 AIC = 1369 vs. Equation 2 AIC = 1333), suggesting that microbial growth kinetics across species scale with temperature in a similar manner to reaction kinetics, but also that growth kinetics can be modified by adaptations allowing rapid translation beyond this baseline expectation (where *Q* is greater than zero; see Supplementary Text S1 for further model analysis and discussion). In Fig. 1a, we see this expectation represented as a dashed line with a negative slope where codon usage bias is zero in our fitted model, such that organisms may have predicted maximum growth rates above this line if their associated value for scaled codon usage bias is above zero. In other words, the relationship with *T*_opt_ sets the baseline for the maximum growth rate, and optimization for faster growth (e.g. via genomic traits increasing the rate of translation) is an independent factor that raises ${\mu}_{\mathrm{max}}$ above this baseline. This result contrasts with within-organism growth kinetics, where the Arrhenius curve does not fit well across the full range of growth temperatures a single organism may encounter [47, 50], because instantaneous growth rates ultimately decline beyond a given species’ *T*_opt_. It is known that an Arrhenius (i.e. exponential) model better captures between-species patterns in temperature optima because each species is adapted for that particular optimum, thus mitigating any impact of protein denaturation or other negative fitness effects that may occur as temperatures increase [21, 51]. See Supplementary Text S1 for further discussion of this model.

Consider the hypothetical scenario where we sample organisms along a temperature gradient where the *T*_opt_ of an organism is inversely related to its codon usage bias. This is potentially a good approximation of microbial populations in the surface ocean, where lower-latitude microbes are adapted to low nutrient supply in warmer waters and high-latitude microbes are adapted to high nutrient supply in colder waters (indeed, in the oceans, productivity is often correlated with cooler water temperatures because it relies on the upwelling of cold, nutrient-rich waters from depth.) In this case, one will likely obtain a series of organisms for which maximum growth rate appears to be negatively correlated with codon usage bias (Fig. 1b and c), despite a positive mechanistic link between these two values encoded in our growth model. We show one example of this in Fig. 1, but this behavior is theoretically observable over a wide range of possible negative relationships between *T*_opt_ and *Q* (Supplementary Text S1). In short, when sampling along a temperature gradient it is possible to reverse the observed relationship between translation optimization and maximum growth rate, even if the overall mechanistic relationship does not change, if *Q* and *T*_opt_ are negatively correlated across organisms.

### Translation optimization is frequently negatively correlated with optimal growth temperature

**Figure 2:**
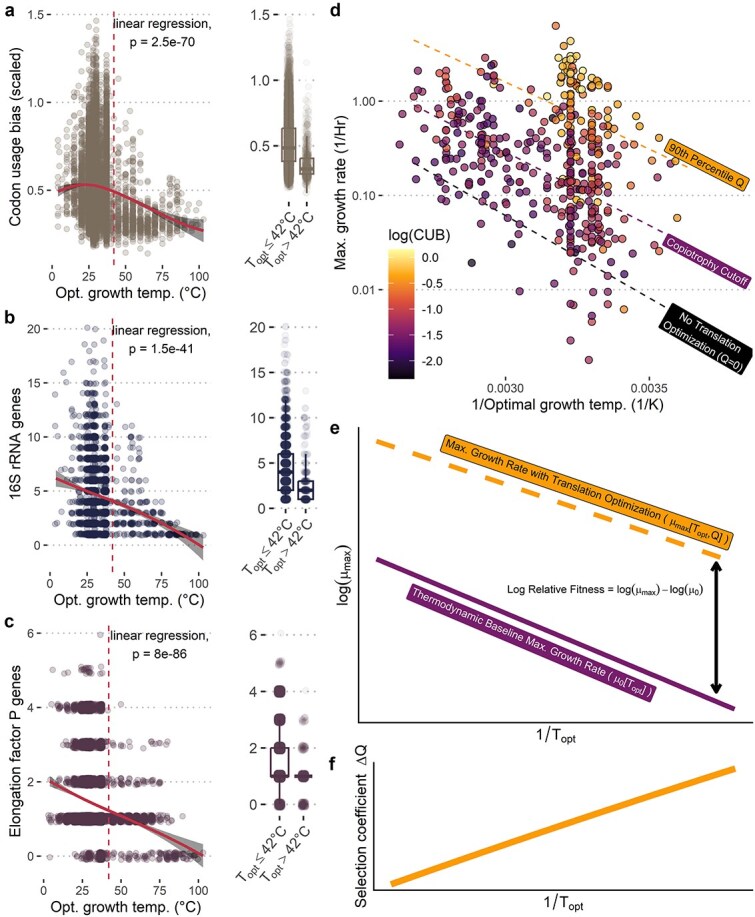
Translation optimization is negatively correlated with optimal growth temperature across species. (a–c) Multiple genomic signatures associated with optimization for rapid translation are negatively correlated with an organism’s *T*_opt_. Smoothing lines depict a GAM fit. Temperature data from a large species-level database of genome-matched *T*_opt_ from the literature (*n* = 12 553). (d) The fit of a diminishing returns model of translation optimization overlaid on data as in Fig. 1a, with dashed lines depicting model fit for different values of codon usage bias (no bias, the theoretical threshold between copiotrophy and oligotrophy as proposed in [6], and the 90th percentile of codon usage bias based on the data shown in this panel). (e and f) Conceptual figures showing how relative fitness is defined under the diminishing returns model and how the selection coefficient for an increase in translation optimization changes with increasing *T*_opt_ under this model.

We analyzed a database of 12 553 genome-matched species’ optimal growth temperatures drawn from the literature, as well as 112 441 genomic predictions of optimal growth temperatures for species representative genomes from GTDB v220 [30, 33, 52]. We found that genomic signatures of translation optimization were less likely to appear at hotter optimal growth temperatures than colder growth temperatures, with psychrophilic and mesophilic organisms showing high levels of translation optimization and thermophiles showing a striking absence of any such signals (Fig. 2a–c and Supplementary Fig. S2). This negative relationship was robust to phylogeny (phylogenetic linear regression; codon usage bias, P = 2.4e-47; rRNA, P = 0.02; EFP, P = 2e-3; Supplementary Fig. S3) and was apparent across a wide variety of habitat types (Supplementary Fig. S4), although was notably absent in host-associated organisms for which temperature ranges tended to be narrower (Supplementary Fig. S5). Nevertheless, the negative translation optimization versus temperature relationship persists even among organisms that live in habitats that never reach high temperatures (e.g. those above ~40°C; Supplementary Fig. S4). This negative relationship was strongest among fast-growing organisms (Supplementary Fig. S2), as there were many slow-growing organisms with little translation optimization present across the range of optimal growth temperatures (Fig. 2a–c).

The cause of the general negative relationship between temperature and translation optimization is not immediately apparent. According to our base Arrhenius model, the relative fitness benefit of increasing translation optimization is constant across temperatures (Supplementary Text S1). We considered three potential hypotheses for why organisms with higher optimal growth temperatures might evolve weaker genomic signals of translation optimization. We first hypothesize that lower effective population sizes (*Ne*) in heat-loving organisms may lead to less efficient selection. Differences in *Ne* have previously been suggested as one reason for the different degrees of codon usage bias observed among psychrophiles, mesophiles, and thermophiles [9]. Genomic signatures like codon usage bias are the result of many mutations with small fitness effect, so that if *s < <1/Ne*, where *s* is the coefficient of selection for one of these small effect mutations and *Ne* is the effective population size, drift might overwhelm the evolution of translation optimization. In general *Ne* is difficult to estimate in microbial populations [53], and is particularly challenging to estimate in thermophiles where commonly used dN/dS based proxy measures of *Ne* likely break down due to the strong purifying selection and low mutation rates experienced by these organisms, possibly resulting from the need to maintain protein function under extremophilic conditions [54–58]. For confident *Ne* estimation, a corresponding estimate of an organism’s mutation rate (typically from a mutation accumulation experiment) is needed. To-date, we know of only one thermophilic organism with an available whole-genome mutation rate estimate and corresponding *Ne* estimate, *Thermococcus eurythermalis* A501 (Ne = 5.83e6, with mutation rate the authors described as “unexpectedly” high [59]), which did have a lower estimated *Ne* than typical of free-living microbes [59]. Low *Ne* in thermophiles is to be expected because their census population sizes are likely lower than those of mesophiles. Habitats that reach temperatures greater than 40°C are less common and frequently are produced by geological (e.g. hot springs) or metabolic (e.g. compost heaps) sources of thermal energy rather than solar- or host-derived heat (Supplementary Fig. S6). Nevertheless, low effective population size alone cannot explain why the negative correlation between *T*_opt_ and translation optimization persists among organisms with *T*_opt_ below 40°C or that live in habitats that never reach that temperature (Supplementary Fig. S4), and the lack of accurate mutation rate data across thermophiles limits our ability to assess this hypothesis systematically.

Our second hypothesis is that constraints on maximum growth rates unrelated to translation optimization could lead to diminishing returns for increased translation optimization as the maximum growth rate of an organism increased, and that this could in turn lead to decreased selection for translation optimization at higher growth temperatures. In other words, the degree of translation optimization may have a smaller impact on maximum growth rates at higher temperatures than lower temperatures. Such a pattern could occur if some other process involved with growth became rate-limiting as translation rates and/or ribosomal investment increase. For example, it has been shown that DNA replication rates increase with temperature more slowly than growth rates in *E. coli*, necessitating multiple replicons [60]. If there is an upper limit on replicon number due to physical constraints in the cell, this would limit the usefulness of translation optimization at high temperatures. A similar argument could be made for any cellular process whose rate scales more slowly with temperature than does translation. We incorporated a diminishing return directly into our base Arrhenius model (Fig. 2d and e; Supplementary Text S1.2), which confirmed that the selection coefficient for increased translation optimization would be inversely related to *T*_opt_ under a diminishing return scenario (Fig. 2f; Supplementary Text S1.2).

**Figure 3:**
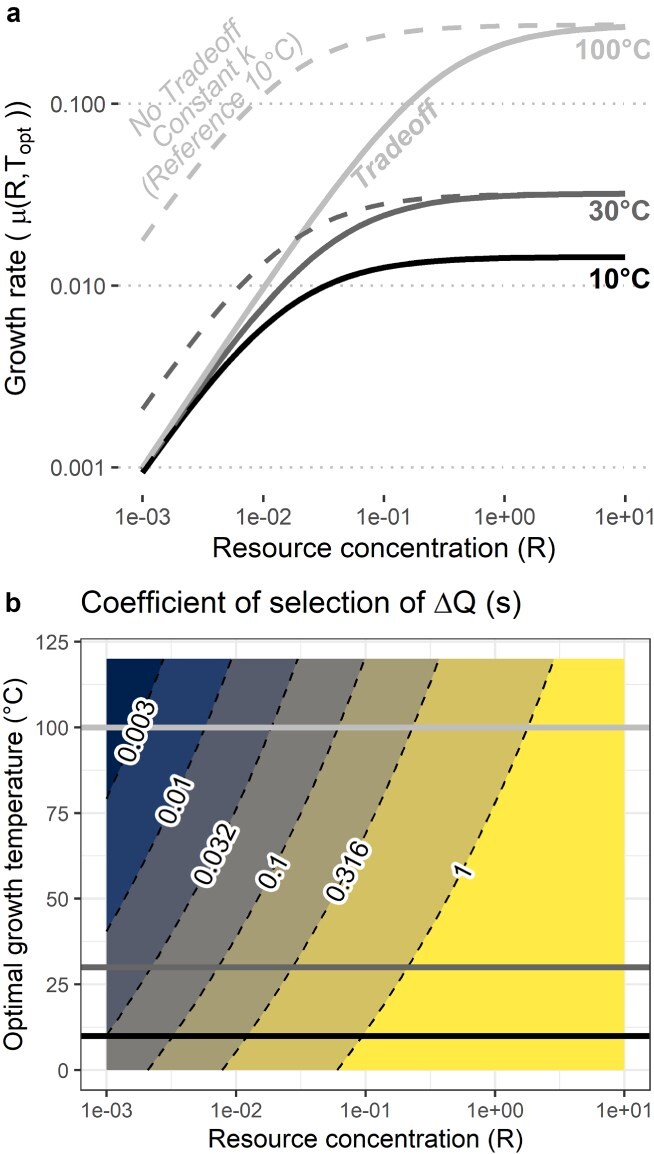
A rate-affinity tradeoff in microbial growth can lead to diminished selection for translation optimization and high growth temperatures and low resource concentrations. (a and b) Conceptual figures showing how growth rates change with resource concentrations under the tradeoff model (model details in Supplementary Text S1.3) and how the selection coefficient for an increase in translation optimization changes with increasing *T*_opt_ and resource concentration under this model. (a) Under our tradeoff model (thick lines), organisms with different optimal growth temperatures will have very different maximum growth rates but will converge on similar rates when resource concentrations are low (*c* = 1, *Q* = 0, and β_i_’s fitted from the base model on gRodon training data). In contrast, without a tradeoff we would see thermophiles with high maximum growth rates grow faster than psychrophiles with lower maximum growth rates at all resource concentrations (dashed lines; assuming a constant half-saturation parameter chosen to be identical to growth at 10°C with a tradeoff for comparison). (b) Under the tradeoff model the coefficient of selection for a mutation increasing translation optimization decreases with both increasing temperature and decreasing resource concentration (*c* = 1, *Q*_WT_ = 0, Δ*Q* = 0.27, and β_i_’s fitted from the base model on gRodon training data; representing the extreme example of selection for a mutation that would increase translation optimization sufficiently to turn an organism with no optimization into a copiotroph; see Supplementary Fig. S7 for an example with a smaller mutation size).

Our third hypothesis is that faster growth itself could simply be less beneficial at higher temperatures, decreasing the selection coefficient for increases in *Q*. In other words, rather than diminishing returns, there may be no evolutionary incentive to optimize for faster growth at these temperatures for other reasons. For example, if thermophiles as a rule experienced a stronger tradeoff between resource affinity and maximum growth rate than mesophiles, this could lead to weaker signals of translation optimization in thermophiles. We modeled a tradeoff between maximum growth rate and resource affinity by embedding our base Arrhenius growth model into a resource-explicit model of instantaneous growth following modified Michaelis–Menten dynamics (Fig. 3; Supplementary Text S1.3). Under this tradeoff, we see that the coefficient of selection for increased translation optimization is inversely related to *T*_opt_ and positively related to local resource concentrations (Fig. 3, Supplementary Fig. S7; Supplementary Text S1.3). In this model resource affinity is linked directly to maximum growth rate, so that fast growth due to either high temperatures or translation optimization can lead to low affinity (such a negative relationship has been shown for extracellular enzymes in leaf litter, possibly owing to fundamental constraints on reaction kinetics [61]). Although our simple model demonstrates the feasibility of one potential mechanism by which a tradeoff could lead to a lack of selection for translation optimization in thermophiles, other tradeoffs could induce a similar effect. For example, some ecological models suggest that slower growth should be favored at warmer temperatures due to tradeoffs with predation rate [62, 63].

It is likely that some combination of our three hypotheses leads to the observed negative correlations between *T*_opt_ and *Q* (Fig. 2a–c), though insufficient data make it challenging to distinguish them at this time.

### Translation optimization and thermophily rarely co-occur in an organism

**Figure 4:**
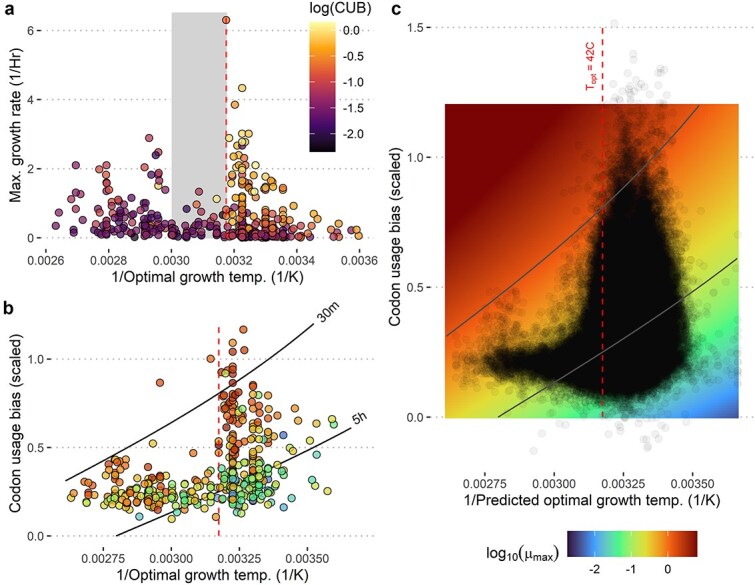
Fast-growing organisms either have high translation optimization or a high optimal growth temperature, but very rarely both. (a) Data as in Fig. 1a, plotted on a linear y-axis. Shaded region denotes the hypothetical thermophile-mesophile gap between 42°C and 60°C where fast-growing organisms are absent from this and other datasets [21, 22]. Vertical dashed line at 42°C. (b) The same dataset plotted on different axes, shows that organisms with *T*_opt_ > 42°C almost never have elevated codon usage bias. Solid lines show contours from the translation optimization diminishing returns growth model (Supplementary Text S1) at a minimum doubling time of 5 h and 30 min. Notice that organisms near the 30 min contour either have high *T*_opt_ or a high codon usage bias, but not both. Color fill shows maximum growth rates from the literature (experimental). (c) Species representative genomes from GTDB v220 mapped onto the space in (b) (*n* = 112 441), with predicted maximum growth rates for each combination of codon usage bias and *T*_opt_ from the diminishing returns model shown as a color gradient in the background (same scale for panels b and c; empirical rates in b and predicted rates in c).

Above a temperature threshold of 42°C, we observed few organisms with signs of translation optimization (Figs. 2, 4 and Supplementary S2). It has previously been observed that there is an absence of fast-growing organisms with *T*_opt_ in the range 42°C–60°C [48], termed the “thermophile-mesophile gap” (Fig. 4a). Viewed alongside information about organismal codon usage bias and our maximum growth rate model, we propose that this gap exists between two kinds of fast-growing organisms. Below 42°C, organisms with fast maximum growth rates are those with high codon usage bias (Fig. 4a and b). Above 42°C, organisms do not have high codon usage bias, but as optimal growth temperatures increase beyond 60°C basic thermodynamics leads to faster growth. The “gap” between these two classes of fast growers is where high optimal growth temperature cannot compensate for low translation optimization. In other words, there are two ways an organism may increase its maximum growth rate—either by increasing its optimal growth temperature or by optimizing its genome for rapid growth (e.g. by increasing the rate of translation of highly expressed genes), but organisms rarely if ever seem to combine these two traits (Fig. 4b and c).

### Translation optimization and maximum growth rate decouple along temperature gradients

**Figure 5:**
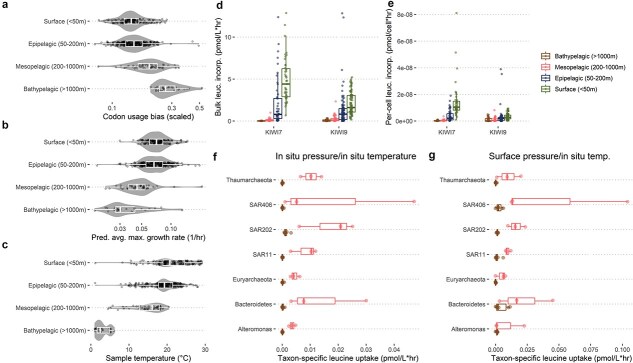
Translation optimization increases with ocean depth, even as maximum and instantaneous microbial growth rates decline into the bathypelagic. (a–c) Average community-wide codon usage bias, sample temperatures, and predicted average maximum growth rates from BIOGEOTRACES and Malaspina metagenomes. We observe that codon usage bias and predicted maximum growth rate are anti-correlated across depths. (d and e) Bulk and per-cell radioactively labeled leucine uptake rates in the Southern Ocean (AESOPS Cruises KIWI7 and KIWI9) decline with depth from the surface through the bathypelagic. (f and g) AESOPS Cruisese uptake rates measured globally (Malaspina Expedition) decline from the mesopelagic to the bathypelagic. This is true when measured at in situ pressures (f) or pressure at the ocean surface (g).

Given the above results that (i) negative *T*_opt_ versus translation optimization relationships can cause misleading negative correlations between translation optimization and maximum growth rates (Fig. 1) and (ii) that such negative relationships are common (Fig. 2a–c, Supplementary S3 and S4), we sought to find examples of these patterns in environmental datasets. For example, looking across depths in the global oceans using metagenomes from the BIOGEOTRACES and Malaspina datasets and temperature-corrected community-level average maximum growth rate predictions from gRodon [38, 39], we found that codon usage bias is strongly elevated at the community level in the deep ocean (>1000 m; Fig. 5a). This result defies common wisdom about marine microbial growth because instantaneous growth rates in the bathypelagic zone are expected to be slow due to the combined effects of temperature, pressure, and nutrient quality [8, 64]. Indeed, due to a strong temperature gradient, temperature-corrected metagenomic estimates of community average maximum growth rate still decrease with depth, even as translation optimization increases (Fig. 5a–c). This apparent decoupling of predicted maximum growth rate and translation optimization aligns with field measurements of instantaneous growth which also decrease with depth (Fig. 5d–g). We obtained ship-based measurements of bacterial production, indicating instantaneous growth, using radioactively labeled leucine incorporation from the Antarctic Environment and Southern Ocean Process Study (AESOPS; [40, 65]) and the Malaspina Expedition [41]. Both studies measured productivity in the deep ocean and either provided per-cell rate measurements or cell counts that allowed for calculation of a per-cell rate. The Malaspina study additionally performed experiments at in situ and surface pressure. Measured bulk and per-cell productivity declined with depth (Fig. 5d–g), in line with general expectations about the deep ocean, even when controlling for the growth-limiting effects of pressure (suggesting a role for temperature in constraining growth; Fig. 5g). Although instantaneous growth rates measured via leucine uptake and maximum growth rates predicted via metagenomics were not sample-matched across these various studies, the global distribution of these sampling expeditions suggest they reflect general patterns of microbial growth along depth gradients. In sum, despite the predicted and observed decrease in microbial growth rates in the deep ocean, genomic signatures of rapid growth (codon usage bias) are strongly elevated in these systems. This suggests that microbial genomes in the bathypelagic are shaped by evolutionary pressures favoring faster growth rates even as the environment constrains observed maximum and instantaneous rates.

Moving beyond marine systems, we also examined a dataset of *streptomyces* sister taxa sampled from a latitudinal gradient of soils across North America [36], and saw a similar pattern where isolates from a Southern phylogroup had warmer *T*_opt_ and decreased codon usage bias relative to a Northern phylogroup, leading to a negative correlation between codon usage bias and maximum growth rate across these groups (Supplementary Fig. S8).

Considering the two paths organisms may take to achieve rapid growth (Fig. 4), we additionally examined biogeographic patterns in marine systems (Supplementary Text S3, Supplementary Fig. S9) and the distributions of functional genes across the tree of life (Supplementary Text S2, Supplementary Figs. S10, S11, S12). We found that biogeographic and functional traits linked to copiotrophic lifestyles are correlated with translation optimization, but not necessarily maximum growth rate itself due to the potential for confounding temperature effects.

## Conclusions

We found that negative relationships between genomic translation optimization and optimal growth temperature are widespread across taxa and environments (Figs. 2, 4, Supplementary Figs. S2–S4), and showed how such relationships can and do lead to a decoupling of genomic traits that enable rapid growth and actual observed maximum growth rates in the presence of strong temperature gradients (Figs. 1, 5, Supplementary Fig. S9). As a result, genomic translation optimization may be a better predictor of canonically “copiotrophic” metabolic traits than maximum growth rate itself looking across sequenced genomes (Figs. S10, S11, S12).

In some environments, it is possible that the inversion of the translation-optimization versus maximum growth relationship leads genomic traits we expect to be associated with “copiotrophy” to become anti-correlated with growth. Considered out of context, this could lead one to propose certain functional relationships between, *for example,* gene abundances and maximum or instantaneous growth rates that were entirely spurious. Similarly, using genomic translation-optimization (e.g. number of 16S rRNA gene copies, codon usage bias) to infer maximum growth rate across a gradient like depth or latitude in marine systems without a temperature correction could lead to a reversal of the true maximum growth rate relationship in one’s inferences. Because instantaneous growth rate also scales with temperature, this confounding effect is as real for instantaneous growth rates measured directly in the field or lab as it is for maximum growth rates predicted from genomes. In both cases, temperature can act as a major confounder when trying to link growth to gene content or ecological role. The most detailed measurements may not tell us much about the composition of ecological strategies in a community if not discussed in the context of local temperature.

Because temperature modulates growth independently of resource acquisition strategy, we suggest reframing copiotrophy as a set of traits that allow an organism to escape from a thermodynamic baseline maximum growth rate. That is, we can think of degrees of copiotrophy as how far away an organism’s maximum growth rate is from its expected maximum growth rate at the same optimal growth temperature in the absence of translation optimization (Fig. 1a, q = 0 line). Conveniently, measures of translation optimization appear to be reliable proxies of this “off-baseline”, suggesting that they are natural measures of copiotrophy. Our previous work showed, at least among marine heterotrophs, carbon lability sets an additional growth baseline from which organisms similarly diverge, as indicated by elevated codon usage bias in highly expressed genes, to take advantage of resource abundance [8]. Together, these results emphasize the importance of interpreting genomic signals of translation optimization like codon usage bias in the context of resource abundance, though other factors like resource quality and temperature may limit growth maxima in entirely orthogonal ways.

We resolve the disconnect between our proposed definition of copiotrophy as divergence from a thermodynamic baseline and classical definitions based on resource availability by focusing on copiotrophy and oligotrophy as adaptations. For example, consider a thermophile living in a resource-replete environment. Based on our analysis, this organism would be unlikely to possess adaptations for rapid resource acquisition and growth, but may nonetheless uptake resources and grow quickly because high temperatures accelerate enzymatic rates and cellular processes. Is this organism a copiotroph? We would argue that it is not, because the organism lacks a cohesive life history strategy comprising specific adaptations to take advantage of its high resource environment (beyond the adaptation of having a high optimal growth temperature). In contrast, if this organism did have specific adaptations comprising a copiotrophic strategy, then by definition it would have higher fitness (i.e. faster growth) in times of resource abundance than an equivalent organism living in the same environment but without those adaptations. This would be observed as the “off-baseline” growth described above. Translation optimization, though not itself representative of all possible adaptations for high-resource environments, appears to be a reliable indicator of whether or not an organism has evolved a copiotrophic life history strategy because such strategies generally rely on rapid exploitation of resources during times of abundance [16], which in turn requires the ability to rapidly produce ribosomes to make proteins.

It is an open question as to why optimal growth temperatures are negatively correlated with translation optimization across organisms. We suggested three hypotheses: (i) that *Ne* and optimal growth temperature may be negatively correlated due to a scarcity of habitats with temperatures exceeding 42°C (Supplementary Fig. S6), leading to less efficient selection in thermophiles, (ii) that the benefit of mutations increasing translation optimization diminishes with increasing optimal growth temperature because the phenotypic effect of these mutations is smaller at faster growth rates (diminishing returns model, Supplementary Text S1, Fig. 2), or (iii) that fast maximum growth may be less beneficial among organisms with high optimal growth temperatures because of other tradeoffs with growth rate that could become stronger with temperature (tradeoff model, Supplementary Text S1, Fig. 3). It is likely that the observed effect is a product of all three hypotheses, with some variation in their relative importance across environments. To test the first hypothesis would require careful mutation accumulation experiments across thermophilic and mesophilic taxa to get confident estimates of *Ne* at a range of environmental temperatures. Hypothesis two could be tested directly in the laboratory with controlled competitions between mutant strains with optimized or de-optimized codon usage bias across a range of temperatures. Hypothesis three encompasses a broad range of possible tradeoffs, but a clear starting point for further investigation would be to demonstrate resource-affinity tradeoffs in organisms grown across a range of temperatures.

We acknowledge that some organisms may fall outside the copiotrophy-oligotrophy framework entirely, or require additional theoretical considerations for their inclusion. Endosymbionts are one possible example of an “edge case,” because their growth traits will emerge from interactions between their own genomically encoded growth traits and those of their host cell.

The arguments we make about rate variation across a temperature gradient, although simple, are also generalizable to any temperature-dependent rate. That is, one might find the highest copy number of a gene that produces a given metabolite in the genome of an organism that lives at the coldest temperatures, rather than in the genome of an organism that produces that metabolite at the fastest rate. Similarly, two organisms may be equally reliant on the production of a metabolite and produce it at the same rate, but if one lives in a colder environment, it may need a higher gene copy number to sustain that production. Large-scale comparative genomic analyses that ignore how temperatures constrain metabolic rates will consistently miss, or even reverse, important gene-function relationships.

## Supplementary Material

Supplementary_material_wrag147

## Data Availability

No data was generated in this manuscript. All data and scripts used to generate analyses and figures available in the Zenodo repository at 10.5281/zenodo.20546706.
