# Supplementary material for: Genomic traits associated with copiotrophy decouple from maximum growth rate predictions along temperature gradients
Source: ISME J. 2026 Jun 9;20(1):wrag147. doi: 10.1093/ismejo/wrag147 (PMC13359058; doi:10.1093/ismejo/wrag147)
Supplement: Supplementary_material_wrag147 [file supplementary_material_wrag147.zip › S2Text_copiotrophy_jlw.pdf]

Supplementary Text 2: Genomic Traits Associated with Copiotrophy Decouple from Maximum Growth Rate Predictions Along Temperature Gradients

JL Weissman, Alexandra Walling, Hugh Ducklow, Emily J. Zakem

Marine Heterotroph Biogeography Primarily Co-Varies with Translation Rate Optimization

Considering the two paths organisms may take to achieve rapid growth (Fig. 4), we asked whether temperature or translation optimization would better predict microbial biogeography. Using data mapping GTDB v220 representative genomes to biogeographic classes across a Pacific Ocean transect [1] we found that biogeographic classes appeared to be primarily arranged along an axis of translation optimization rather than T_{opt} (Fig. S8). This suggests that by grouping microbes by their maximum or instantaneous growth rate in the presence of strong and confounding temperature gradients we may lose information about the evolved growth strategies of those organisms.

Metabolic Traits Primarily Co-Vary with Translation Rate Optimization

Given the two paths organisms may take to achieve rapid growth (Fig. 4), we predicted that functional genes related to copiotrophy (*e.g.*, those facilitating rapid acquisition of resources) would be most beneficial to organisms that achieve fast maximum growth rates through translation optimization and would be proportionally less beneficial for organisms already capable of rapid growth by nature of life in a warm environment. That is, the benefit of carrying genes allowing for the rapid exploitation of resources would be seen most dramatically in growth-rate maximizers who are also translation-rate optimizers but would be minimal for growth-rate maximizers who simply live in warmer environments.

We built linear models of gene family presence/absence for family-level representative genomes from GTDB v220 using codon usage bias and T_{opt} as predictors (Fig S10, S11, S12). Across models showing significant interactions ($p<0.01$ for both coefficients after

Benjamini-Hochberg correction), we found that most gene families (56%) were positively associated with codon usage bias and negatively associated with T_{opt}, corresponding to growth-maximizers who are also translation-optimizers but not temperature-optimizers (Fig S10). In contrast, only a small minority of gene families (16%) were found to be positively associated with growth-increasing traits in general (positive coefficients for both codon usage bias and T_{opt}). Whereas in most cases gene families with significant coefficients were of unknown or only generally annotated functions, gene families that had a positive codon usage bias relationship and a negative T_{opt} relationship were enriched for functions involved in translation, amino acid transport and metabolism, and ion transport and metabolism, among others (Fig. S10). We repeated the same analysis at the level of metabolic pathways, and found that the presence of metabolic pathways often conceptually associated with copiotrophy such as carbohydrate and amino acid metabolism were positively associated with codon usage bias and negatively associated with T_{opt}, whereas canonically oligotrophic pathways like methanogenesis and hydrogen oxidation are negatively associated with codon usage bias and positively associated with T_{opt} (Figs. S10 and S11). Together, these results suggest that copiotrophic traits are correlated with translation optimization, but not necessarily maximum growth rate itself due to the potential for confounding temperature effects.

Methods

To analyze microbial biogeography we used the mapping of 1,417 GTDB v220 representative genomes to marine biogeographic categories described in Zakem et al. [1] (Fig. 4d-g).

Gene and pathway analyses were performed by building per-gene or per-pathway logistic regression models (glm in R v4.3.3 base stats [2]) with codon usage bias and optimal growth temperature as predictors and correcting for multiple testing using a Benjamini-Hochberg correction.

For all bioinformatics analyses, default parameters were used unless otherwise specified. All data and scripts used to generate analyses and figures available at https://github.com/jlw-ecoevo/copio_temp/.

1. Zakem EJ, McNichol J, Weissman JL, et al (2025) Functional biogeography of marine microbial heterotrophs. Science 388:eado5323. https://doi.org/10.1126/science.ado5323

2. R Core Team (2024) R: A Language and Environment for Statistical Computing. R Foundation for Statistical Computing, Vienna, Austria
