# Supplementary material for: Genomic traits associated with copiotrophy decouple from maximum growth rate predictions along temperature gradients
Source: ISME J. 2026 Jun 9;20(1):wrag147. doi: 10.1093/ismejo/wrag147 (PMC13359058; doi:10.1093/ismejo/wrag147)
Supplement: Supplementary_material_wrag147 [file supplementary_material_wrag147.zip › SupplementalFigures_copiotrophy_jlw.pdf]

Supplementary Figures: Genomic Traits Associated with Copiotrophy Decouple from Maximum Growth Rate Predictions Along Temperature Gradients

JL Weissman, Alexandra Walling, Hugh Ducklow, Emily J. Zakem

Supplemental Figure 1: (a-c) Maximum growth rate is positively associated with various measures of translation optimization, but organisms with high T_{opt} defy this pattern. Solid lines depict GAM fit for organisms with T_{opt} below 42C. (d-f) Organisms with high T_{opt} rarely show signs of translation optimization on their genomes. Dashed red line at 42C. 4
Supplemental Figure 2: The negative relationship between optimal growth temperature and translation optimization is strongest among fast-growing organisms. 5
Supplemental Figure 3: The negative relationship between optimal growth temperature and codon usage bias is robust to phylogeny. (a) Phylogenetic linear regression sensitivity analysis to held-out species. (b) Phylogenetic linear regression sensitivity analysis to held-out phyla. (c) Phylogenetic linear regression sensitivity analysis to varying percentages of the dataset being held-out. (d) The relationship between codon usage bias and T_{opt} for different phyla either using predicted T_{opt} across all of GTDB v220 (black) or a database of growth temperatures from the literature (red). Phyla with at least 500 representatives in GTDB shown. 6
Supplemental Figure 4: The negative relationship between optimal growth temperature and codon usage bias is apparent across diverse environments. Data in panel (a) from a database of T_{opt} measurements from the literature and in panel (habitats with >100 representatives shown) and in (b) from predicted T_{opt} for GTDB v220 (habitats with >250 representatives shown). Habitat data taken from NCBI isolation source information, excluding host-associated habitats (see Fig S5). 7
Supplemental Figure 5: The negative relationship between optimal growth temperature and codon usage bias does not consistently appear among host-associated environments. Data in panel (a) from a database of T_{opt} measurements from the literature and in panel (habitats with >100 representatives shown) and in (b) from predicted T_{opt} for GTDB v220 (habitats with >250 representatives shown)). Habitat data taken from NCBI isolation source information. 8
Supplemental Figure 6: Habitats with temperatures greater than 42C are rare. (a) Distribution of global land surface temperature measurements between -10C and 100C

during four months of 2024 from NASA Terra/MODIS. (b) Distribution of global sea surface temperature measurements between -10C and 100C during four months of 2024 from NASA Aqua/MODIS. (c) Distribution of average animal body temperature measurements across species. Most high temperatures are associated with passerine birds. 9

Supplemental Figure 7: Mutations of smaller effect than those seen in Fig 3 also show similar patterns in their coefficient of selection under the growth tradeoff model. By increasing the strength of the tradeoff (c=100) in this example, we see that even for mutations of small effect, selection will be considerable (3%) for mesophiles and psychrophiles in resource rich environments, but that the strength of selection on translation optimization decreases for thermophiles and/or organisms in resource-poor environments. 10

Supplemental Figure 8: Southern- and nothern-derived phylogroups of streptomyces collected from North America show anti-correlated codon usage bias and optimal growth temperature, as well as anti-correlated codon usage bias and predicted maximum growth rate. Significance levels shown from Welch's t-test. Optimal growth temperatures predicted with GenomeSPOT and growth rates predicted with gRodon...........................12

Supplemental Figure 9: Biogeography for marine picoheterotrophs appears to organize along a gradient of translation optimization (CUB) rather than temperature. (a-d) Data taken from [8] where genomes from GTDB v220 were mapped to biogeographic categories along a transect of the Pacific Ocean running from 60°N to 60°S with depth profiles up to 600 m using high-resolution 16S rRNA gene profiles. (a) Each point is a genome, with color representing the assigned biogeographic category, and ellipses are drawn at the 50th percentile for each category with centroids shown as larger outlined points. Solid black lines show contours of the predicted min. doubling times associated with each estimated growth temperature and codon usage bias. (b-d) The marginal distributions of the categories in panel (as) for the three axes shown in that panel.......................................11

Supplemental Figure 10: Genomic translation optimization predicts the presence of metabolic pathways associated with copiotrophy. (a) The functional breakdown of CUB and OGT associated gene families. Labels on x-axis indicate whether there was a positive or negative association with translation optimization (Q) and optimal growth temperature (T_{opt}), corresponding to the categories in S9 Fig. Notably, the large majority of genes have unknown function or only a general functional prediction, whereas genes families that had positive relationships with translation optimization and negative relationships with temperature (i.e., being primarily translation associated rather than being correlated with max. growth rate) are more likely to be involved in translation, transport, and biogenesis. (b-e) At the pathway level, canonically copiotrophic pathways like carbohydrate and amino acid metabolism tend to be positively associated with codon usage bias, whereas

canonically oligotrophic metabolisms like methanogenesis and hydrogen redox are positively associated with growth temperature ($p<0.01$, Benjamini-Hochberg correction). 13
Supplemental Figure 11: Pathways associated with codon usage bias and/or optimal growth temperatures not shown in Fig 5. 14
Supplemental Figure 12: (a) More gene families correlate with translation optimization (CUB) than maximum growth rate itself across families in GTDB v220. The coefficients of regression models of gene family (COG) presence/absence across genomes where codon usage bias and T_{opt} are significant predictors ($p<0.01$, Benjamini-Hochberg correction) show that most gene families are positively associated with codon usage bias and negatively associated with T_{opt} (category I), whereas only about 16% of the gene family models with significant relationships show a positive association with growth writ-large (category III; i.e., a positive association with both codon usage bias and growth temperature). (b-e) The distribution of CUB and OGT associated gene families across GTDB representative genomes. 15

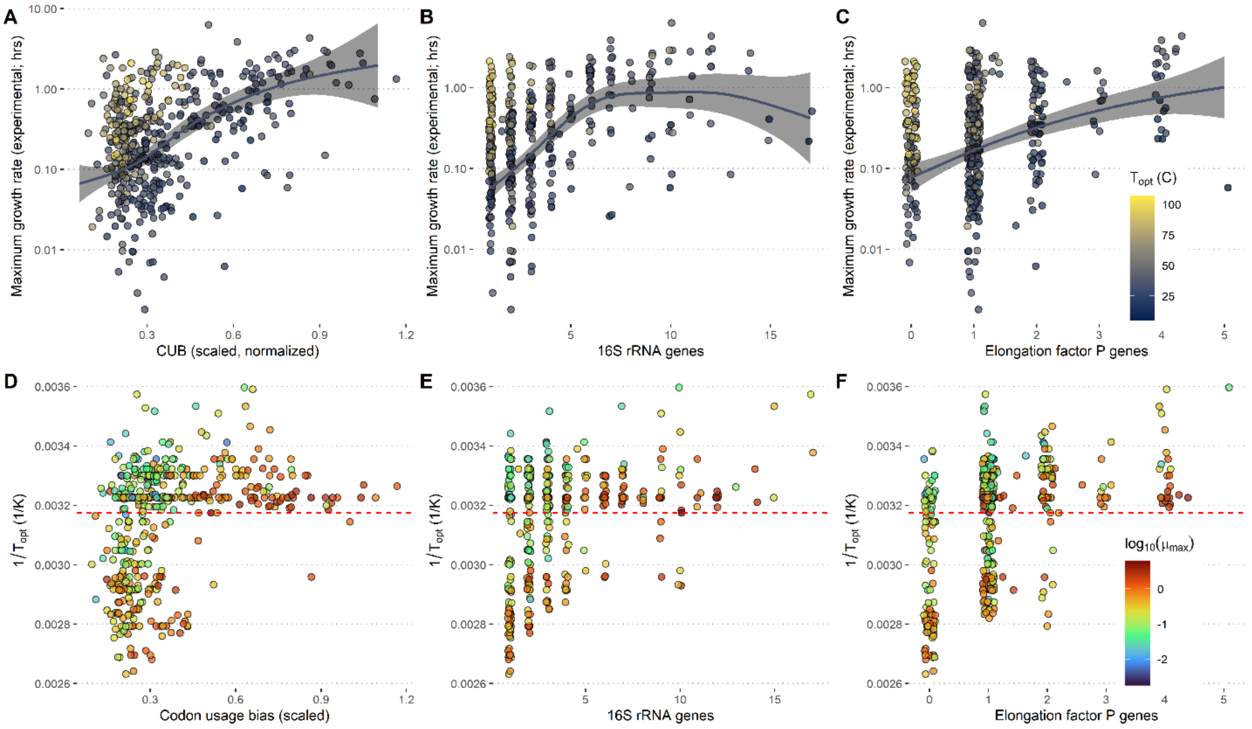

Supplemental Figure 1: *(a-c) Maximum growth rate is positively associated with various measures of translation optimization, but organisms with high* T_{opt} *defy this pattern. Solid lines depict GAM fit for organisms with* T_{opt} *below 42C. (d-f) Organisms with high* T_{opt} *rarely show signs of translation optimization on their genomes. Dashed red line at 42C.*

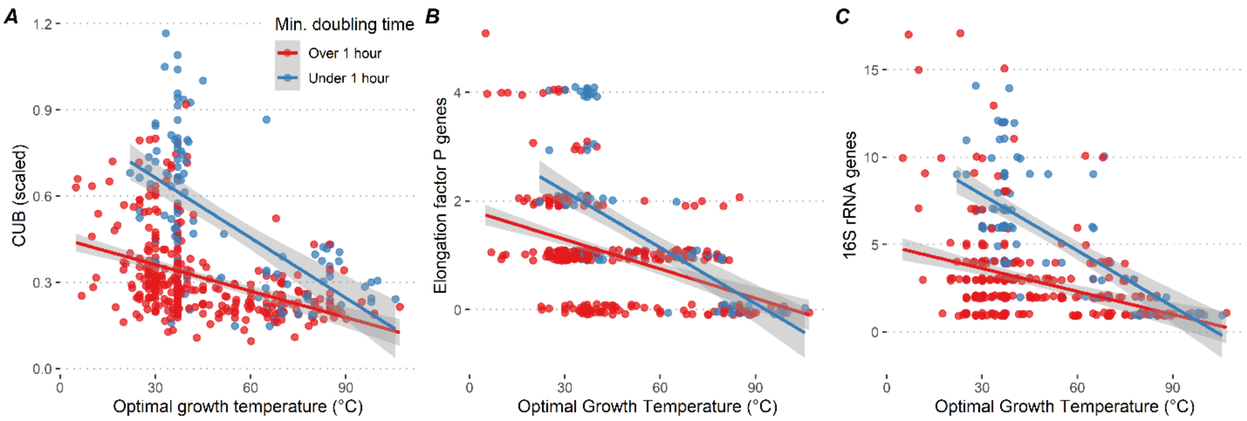

Supplemental Figure 2: *The negative relationship between optimal growth temperature and translation optimization is strongest among fast-growing organisms.*

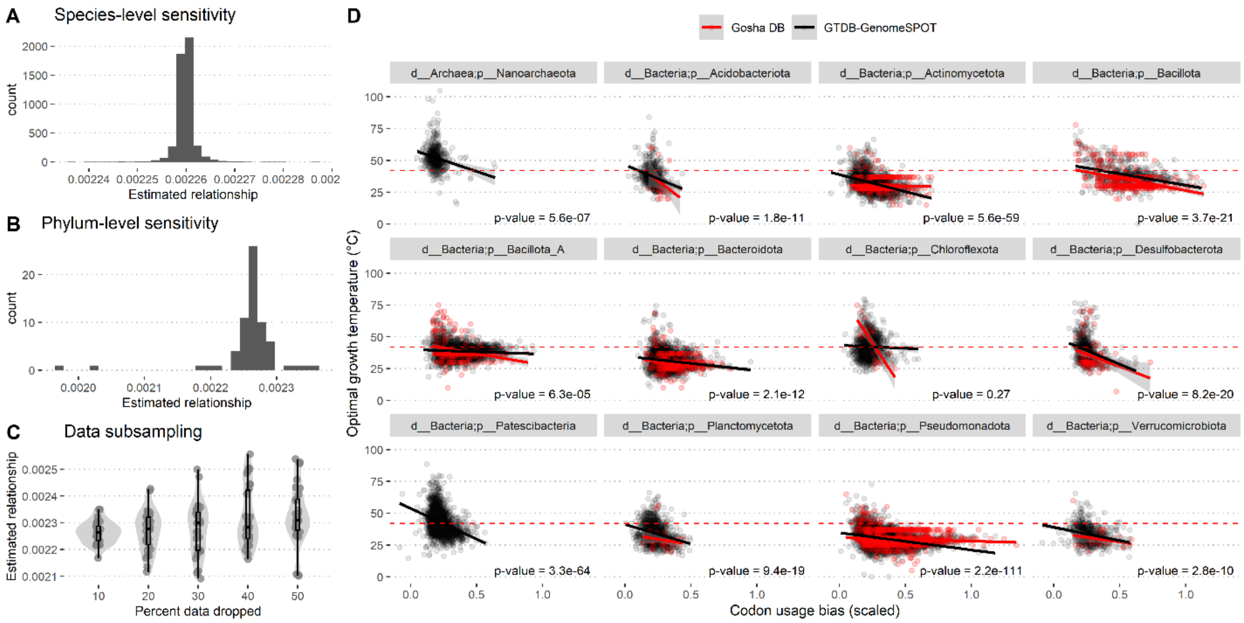

Supplemental Figure 3: *The negative relationship between optimal growth temperature and codon usage bias is robust to phylogeny. (a) Phylogenetic linear regression sensitivity analysis to held-out species. (b) Phylogenetic linear regression sensitivity analysis to held-out phyla. (c) Phylogenetic linear regression sensitivity analysis to varying percentages of the dataset being held-out. (d) The relationship between codon usage bias and* T_{opt} *for different phyla either using predicted* T_{opt} *across all of GTDB v220 (black) or a database of growth temperatures from the literature (red). Phyla with at least 500 representatives in GTDB shown.*

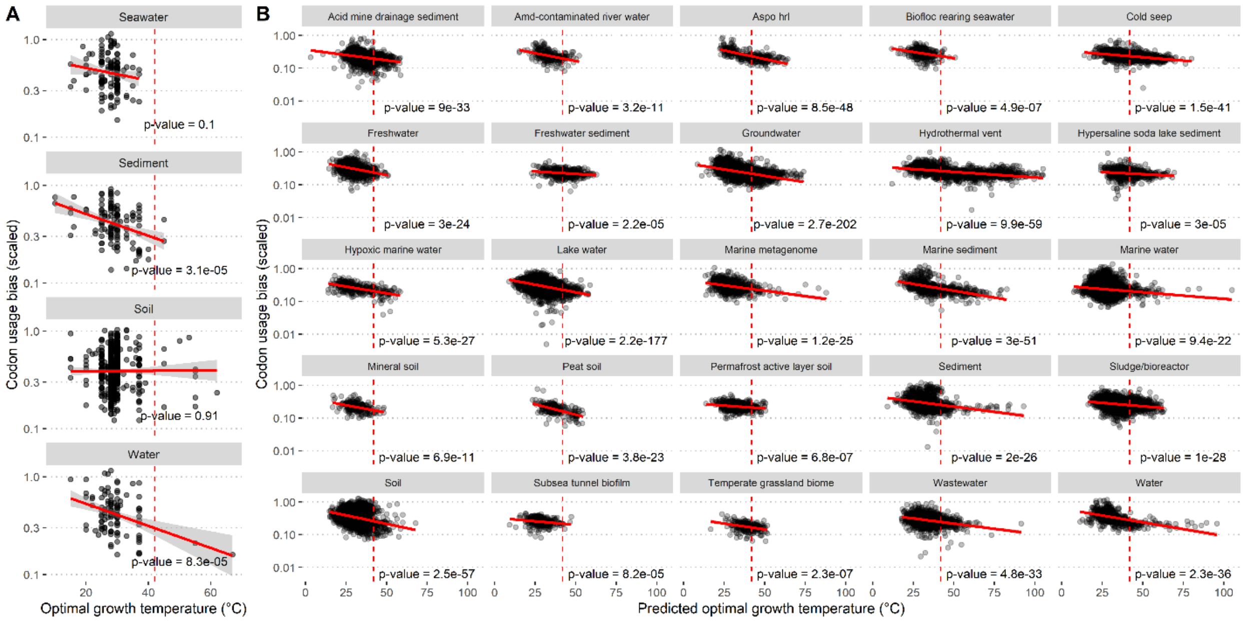

Supplemental Figure 4: *The negative relationship between optimal growth temperature and codon usage bias is apparent across diverse environments. Data in panel (a) from a database of* T_{opt} *measurements from the literature and in panel (habitats with >100 representatives shown) and in (b) from predicted* T_{opt} *for GTDB v220 (habitats with >250 representatives shown). Habitat data taken from NCBI isolation source information, excluding host-associated habitats (see Fig S5).*

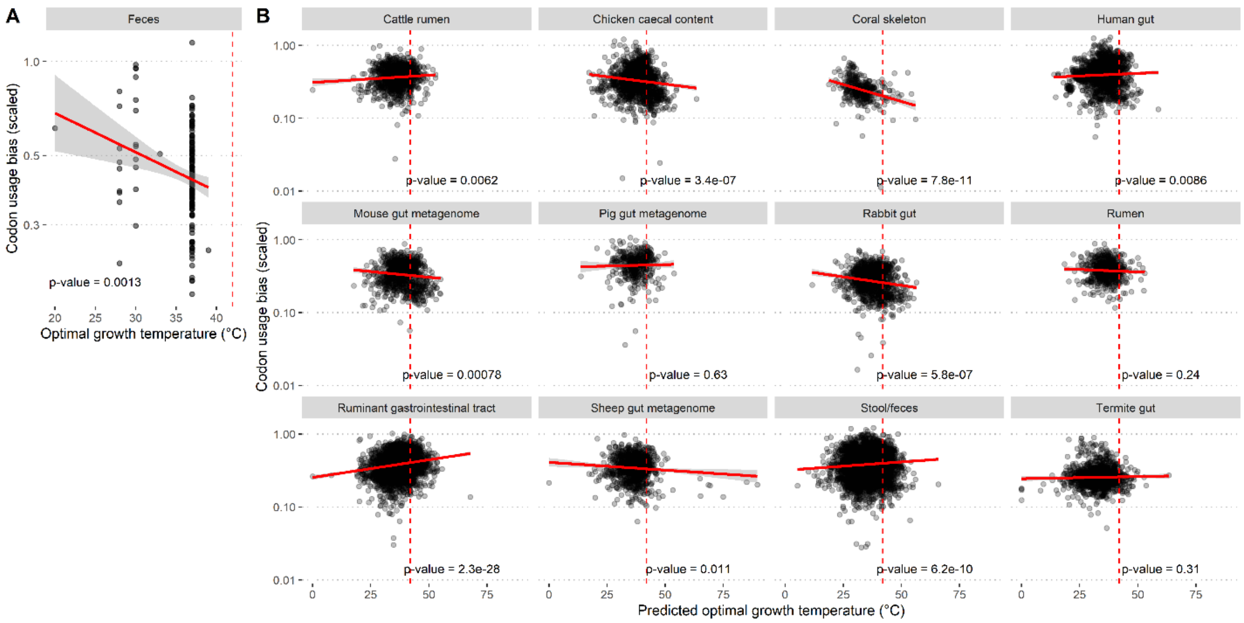

Supplemental Figure 5: *The negative relationship between optimal growth temperature and codon usage bias does not consistently appear among host-associated environments. Data in panel (a) from a database of* T_{opt} *measurements from the literature and in panel (habitats with >100 representatives shown) and in (b) from predicted* T_{opt} *for GTDB v220 (habitats with >250 representatives shown)). Habitat data taken from NCBI isolation source information.*

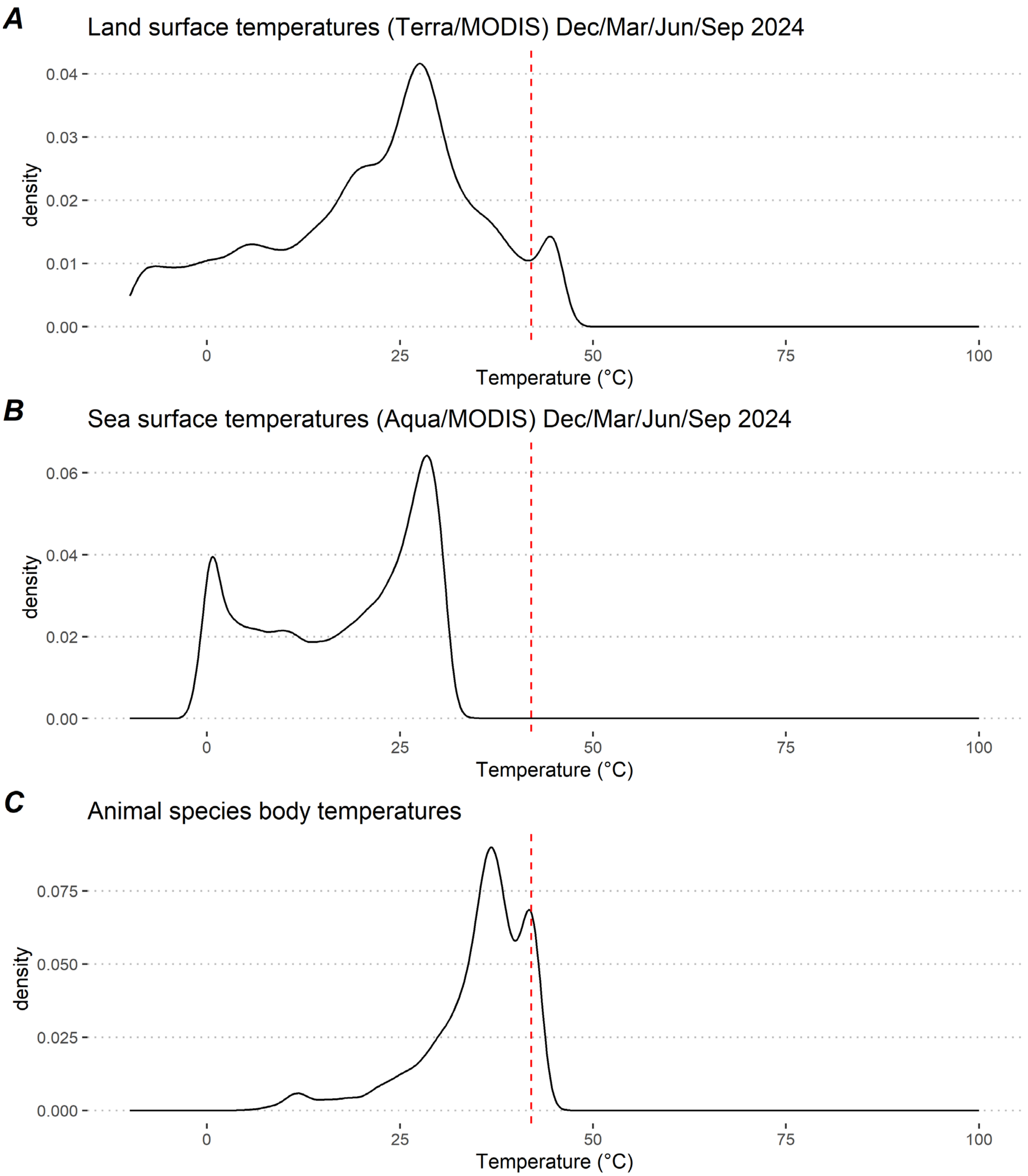

Supplemental Figure 6: *Habitats with temperatures greater than 42C are rare. (a) Distribution of global land surface temperature measurements between -10C and 100C during four months of 2024 from NASA Terra/MODIS. (b) Distribution of global sea surface temperature measurements between -10C and 100C during four months of 2024 from NASA Aqua/MODIS. (c) Distribution of average animal body temperature measurements across species. Most high temperatures are associated with passerine birds.*

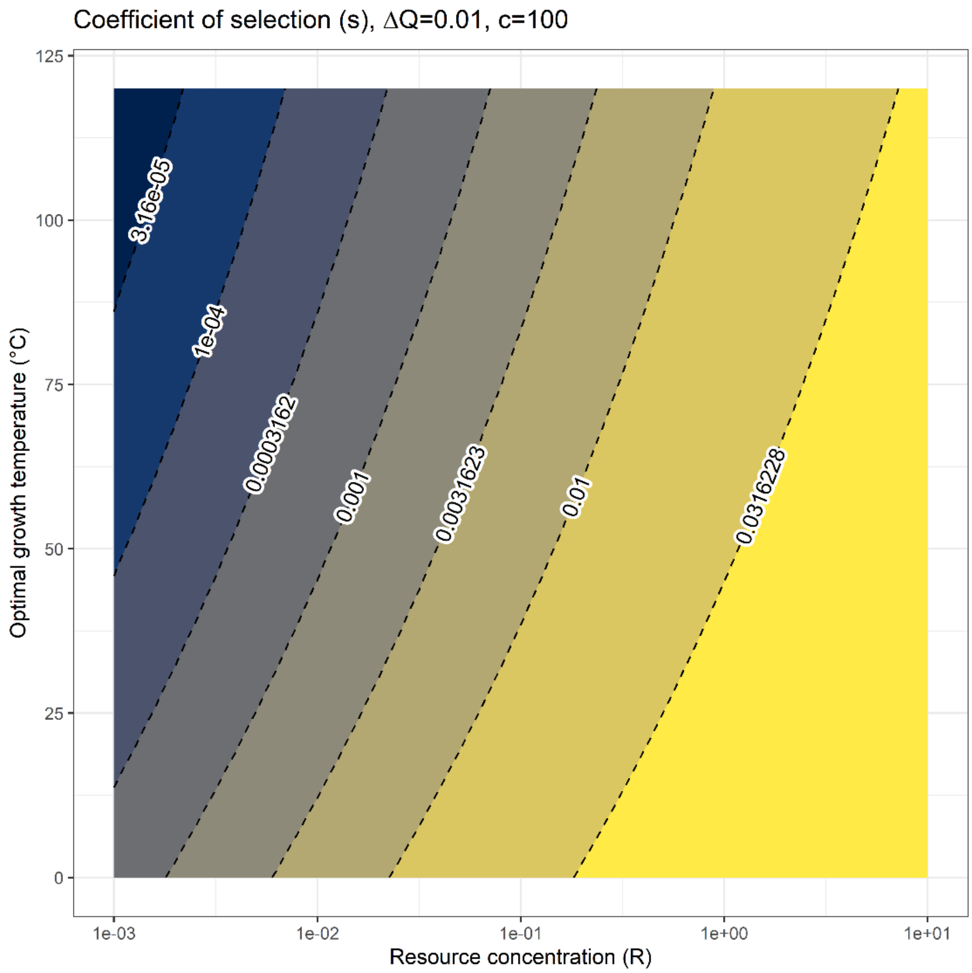

Supplemental Figure 7: *Mutations of smaller effect than those seen in Fig 3 also show similar patterns in their coefficient of selection under the growth tradeoff model. By increasing the strength of the tradeoff (c=100) in this example, we see that even for mutations of small effect, selection will be considerable (3%) for mesophiles and psychrophiles in resource rich environments, but that the strength of selection on translation optimization decreases for thermophiles and/or organisms in resource-poor environments.*

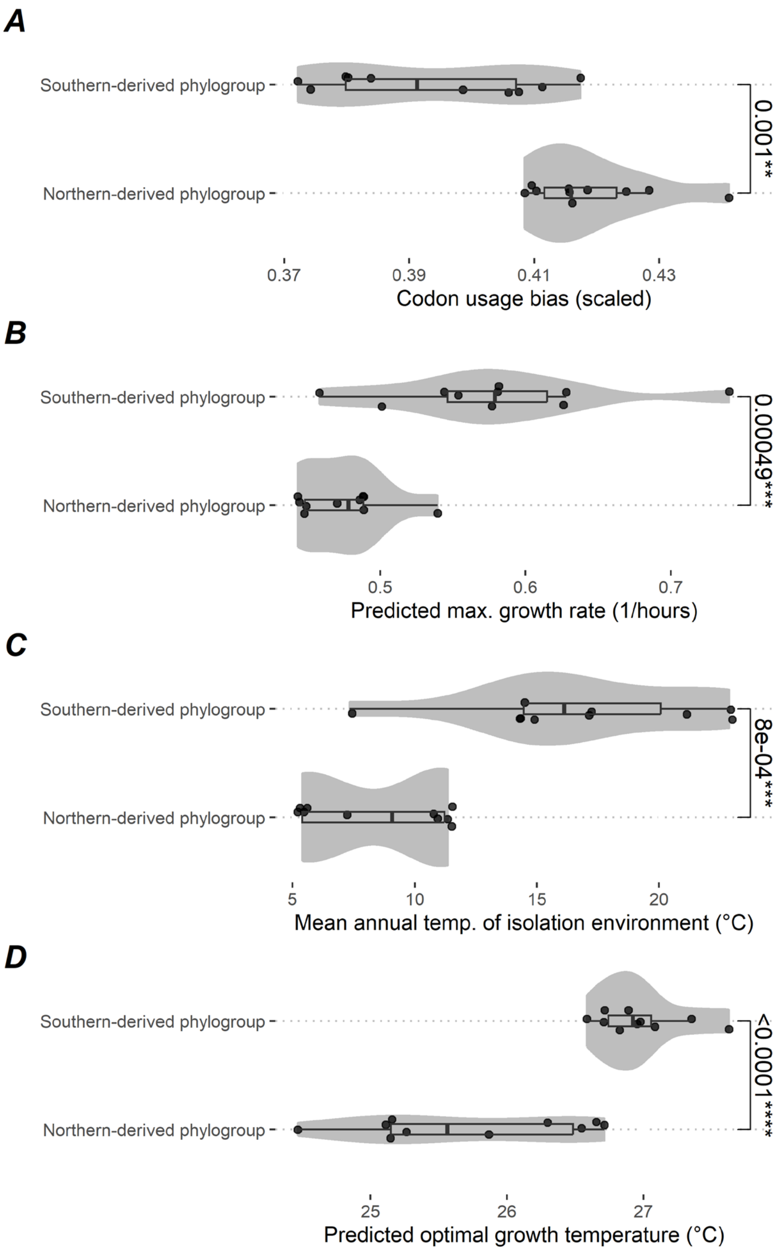

Supplemental Figure 8: *Southern- and nothern-derived phylogroups of* streptomyces *collected from North America show anti-correlated codon usage bias and optimal growth temperature, as well as anti-correlated codon usage bias and predicted maximum growth rate. Significance levels shown from Welch's t-test. Optimal growth temperatures predicted with GenomeSPOT and growth rates predicted with gRodon.*

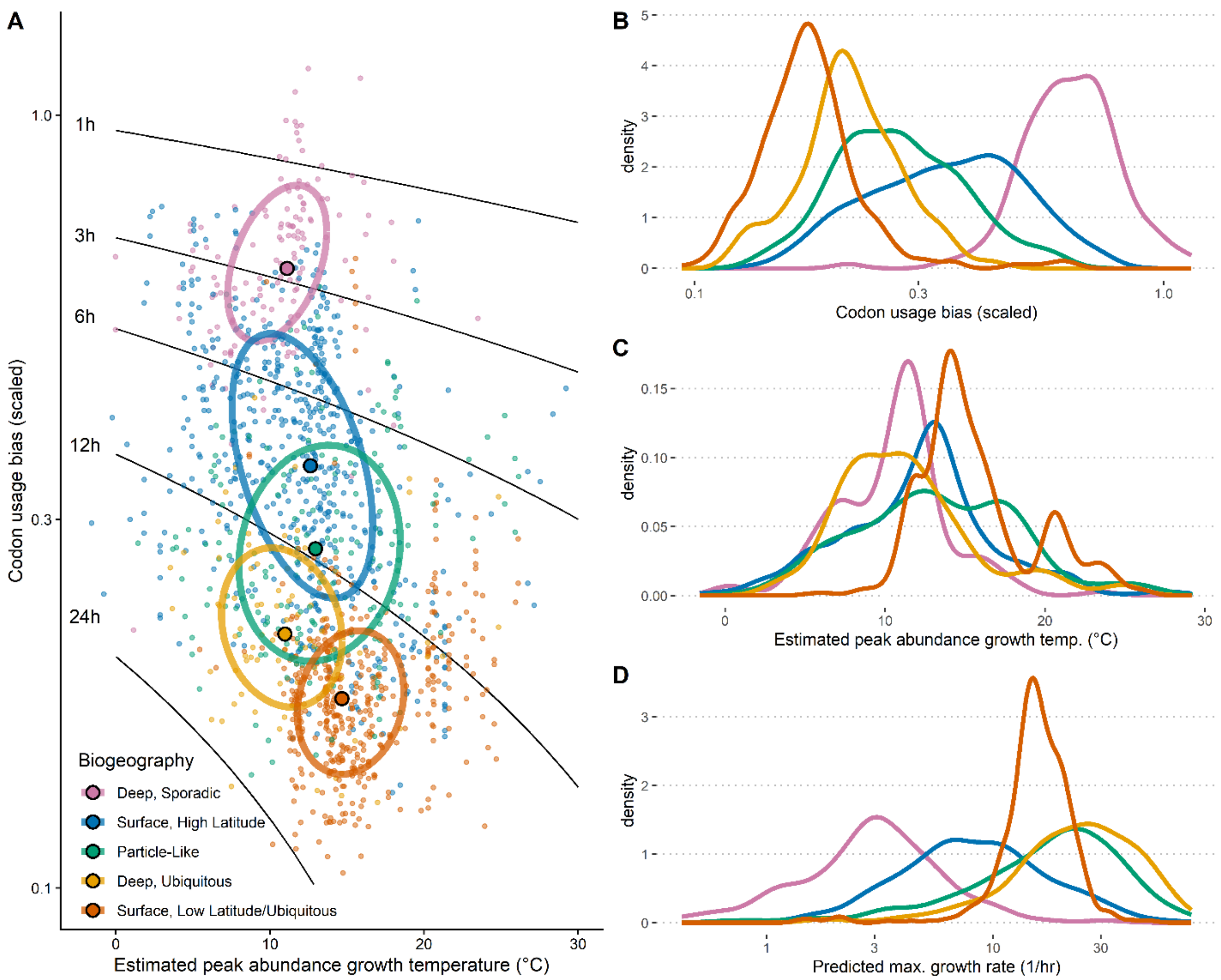

Supplemental Figure 9: *Biogeography for marine picoheterotrophs appears to organize along a gradient of translation optimization (CUB) rather than temperature. (a-d) Data taken from [8] where genomes from GTDB v220 were mapped to biogeographic categories along a transect of the Pacific Ocean running from 60°N to 60°S with depth profiles up to 600 m using high-resolution 16S rRNA gene profiles. (a) Each point is a genome, with color representing the assigned biogeographic category, and ellipses are drawn at the 50th percentile for each category with centroids shown as larger outlined points. Solid black lines show contours of the predicted min. doubling times associated with each estimated growth temperature and codon usage bias. (b-d) The marginal distributions of the categories in panel (as) for the three axes shown in that panel.*

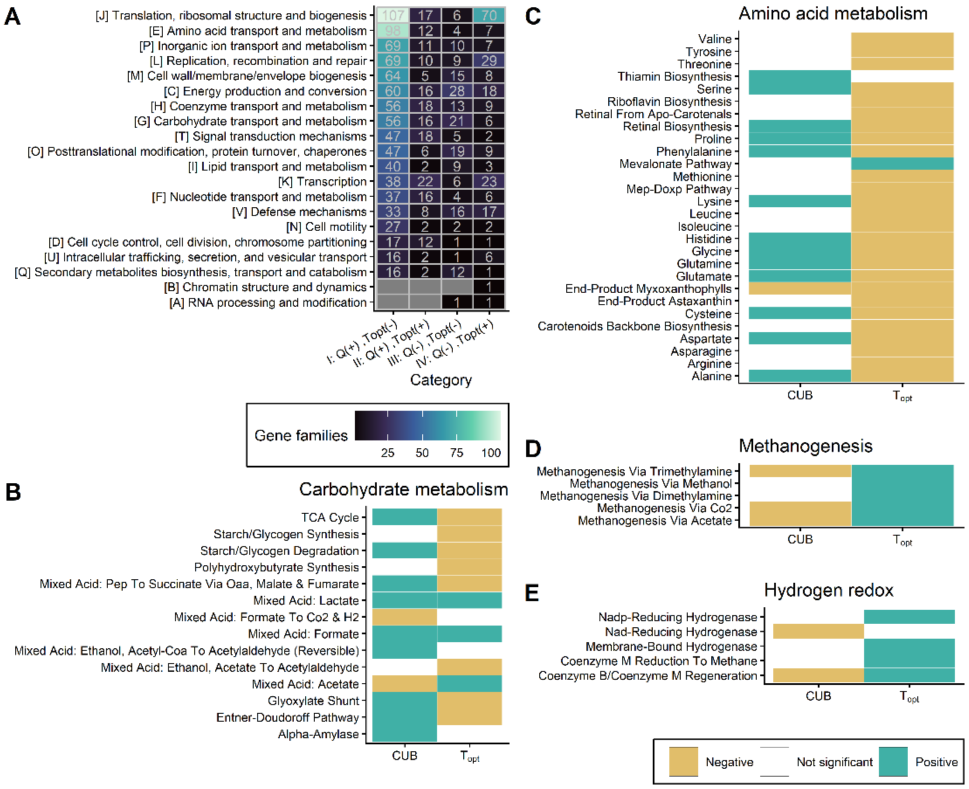

Supplemental Figure 10: *Genomic translation optimization predicts the presence of metabolic pathways associated with copiotrophy. (a) The functional breakdown of CUB and OGT associated gene families. Labels on x-axis indicate whether there was a positive or negative association with translation optimization (Q) and optimal growth temperature (T_{opt}), corresponding to the categories in S9 Fig. Notably, the large majority of genes have unknown function or only a general functional prediction, whereas genes families that had positive relationships with translation optimization and negative relationships with temperature (i.e., being primarily translation associated rather than being correlated with max. growth rate) are more likely to be involved in translation, transport, and biogenesis. (b-e) At the pathway level, canonically copiotrophic pathways like carbohydrate and amino acid metabolism tend to be positively associated with codon usage bias, whereas canonically oligotrophic metabolisms like methanogenesis and hydrogen redox are positively associated with growth temperature ($p<0.01$, Benjamini-Hochberg correction).*

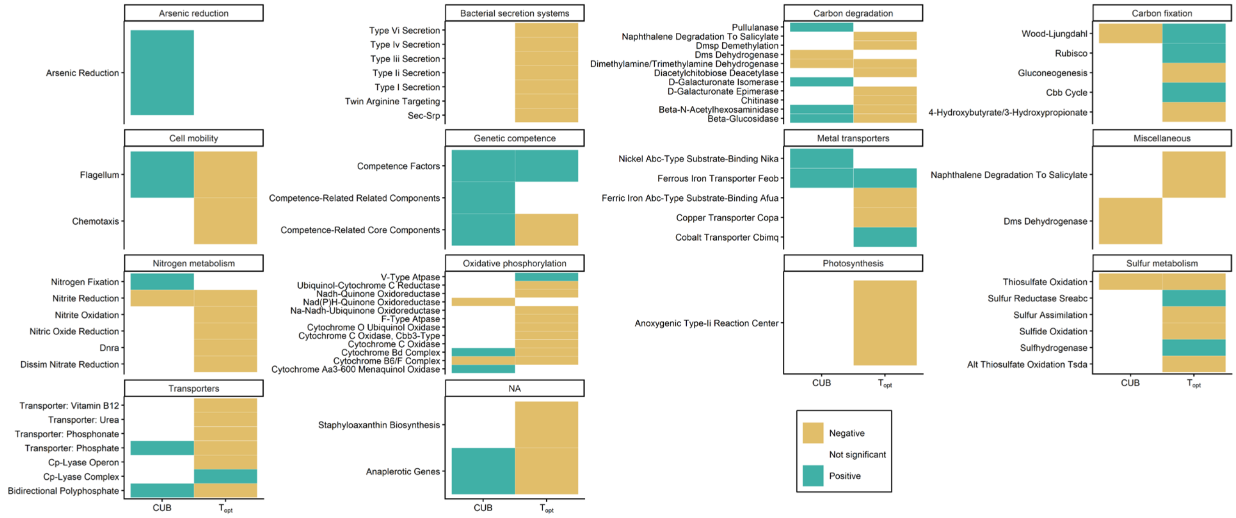

Supplemental Figure 11: *Pathways associated with codon usage bias and/or optimal growth temperatures not shown in Fig 5.*

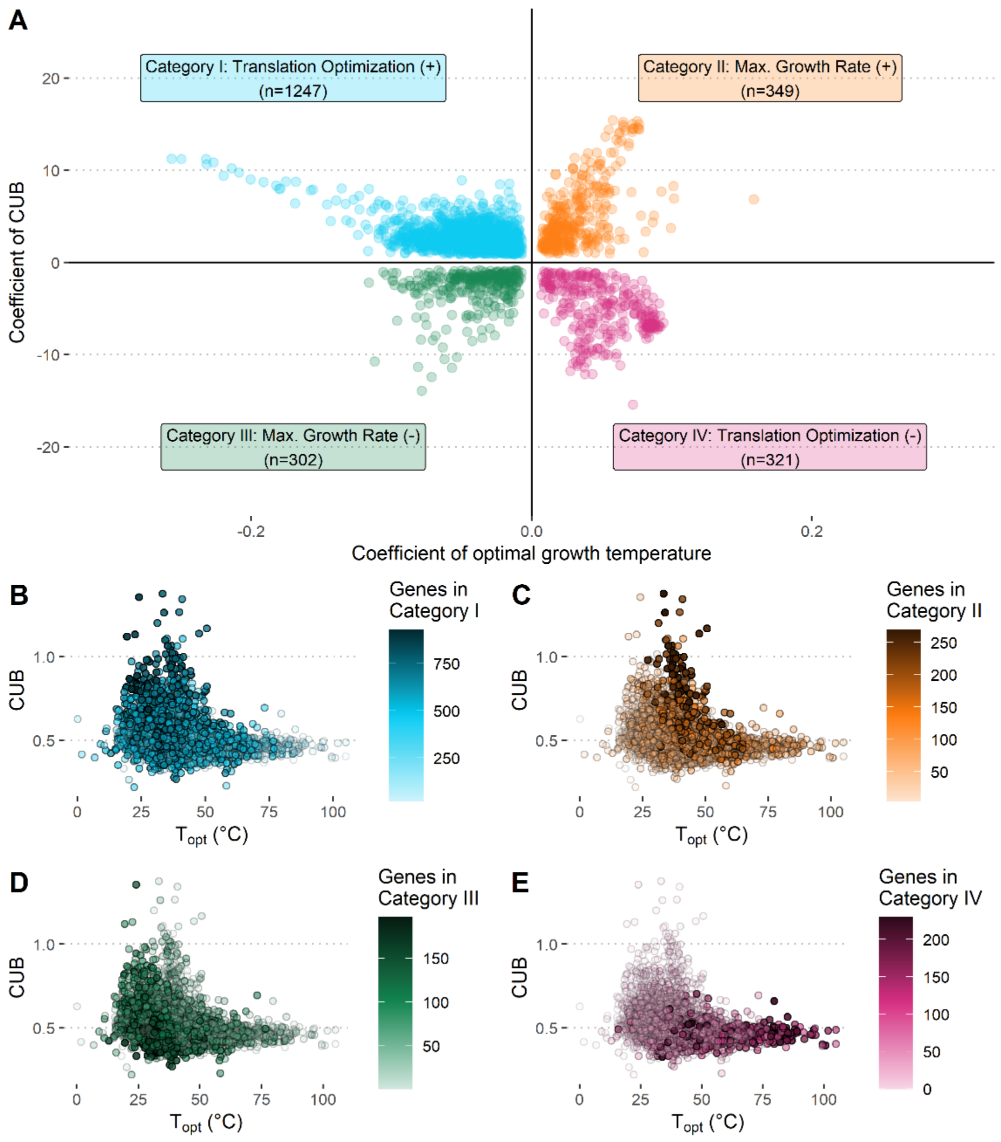

Supplemental Figure 12: *(a) More gene families correlate with translation optimization (CUB) than maximum growth rate itself across families in GTDB v220. The coefficients of regression models of gene family (COG) presence/absence across genomes where codon usage bias and T_{opt} are significant predictors (p<0.01, Benjamini-Hochberg correction) show that most gene families are positively associated with codon usage bias and negatively associated with T_{opt} (category I), whereas only about 16% of the gene family models with significant relationships show a positive association with growth writ-large (category III; i.e., a positive association with both codon usage bias and growth temperature). (b-e) The distribution of CUB and OGT associated gene families across GTDB representative genomes.*
